# Deletion of *Axin1* in aggrecan-expressing cells leads to growth plate cartilage defects in adult mice

**DOI:** 10.1016/j.gendis.2023.101147

**Published:** 2023-10-19

**Authors:** Dan Yi, Hongting Jin, William W. Lu, Chunxiang Zhang, Guozhi Xiao, Liping Tong, Di Chen

**Affiliations:** a Research Center for Computer-aided Drug Discovery, Shenzhen Institute of Advanced Technology, Chinese Academy of Sciences, Shenzhen, Guangdong 518055, China; b Faculty of Pharmaceutical Sciences, Shenzhen Institute of Advanced Technology, Chinese Academy of Sciences, Shenzhen, Guangdong 518055, China; c The First College of Clinical Medicine, Zhejiang Chinese Medical University, Hangzhou, Zhejiang 310053, China; d Department of Cardiology, Basic Medicine Innovation Center for Cardiometabolic Diseases of Ministry of Education, Institute of Cardiovascular Research, The Affiliated Hospital, Southwest Medical University, Luzhou, Sichuan 646000, China; e School of Medicine, Southern University of Science and Technology, Shenzhen, Guangdong 518055, China

In mammals, skeletal bone development begins at hyaline cartilage formation at the early embryonic stage.^1^ Then osteoblasts from the periosteum, which are distributed surrounding the hyaline cartilage, build up compact bone to form diaphysis and spongy bone known as primary ossification center.^1^ Next, osteoclasts from the hematopoietic system destroy spongy bone to generate a medullary cavity.^1^ Along with this continuous establishment of the medullary cavity, after birth, there is a formation of a secondary ossification center occurring at both polar sites (epiphysis) of original hyaline cartilage in a post–axis manner.^1^, ^2^, ^3^, ^4^ After the formation of the secondary ossification center, the cartilage will be substituted by the spongy bone but leave intact articular cartilage and growth plate (epiphyseal plate) which is comprised of chondrocytes.^1^ This process is known as endochondral ossification.^1^ During endochondral ossification, the growth plate exerts a fundamental role in increasing the length of the skeletal bone.^1^ There are three principal layers of the structure of a growth plate: resting zone, proliferating zone, and hypertrophic zone.^2^ The cells with mesenchymal stem cell features in the resting zone give rise to clones of proliferating chondrocytes and control the alignment of the proliferating chondrocytes into columns parallel to the long axis of the bone via producing morphogens.^2^ Proliferating zone is responsible for elongating endochondral bone shape by dividing and arranging clones of chondrocytes into columnar structure.^2^ Chondrocytes in the hypertrophic zone generated by terminal differentiation of proliferating chondrocytes are contributing to long bone growth by attracting vascular and progenitor cell invasion from adjacent areas.^2^ Sustained chondrocyte proliferation and hypertrophy lead to calcification and accumulation of bone, thus lengthening the bone during skeletal development and postnatal bone growth.^1^^,^^2^ Unlike humans, there is no termination of proliferation and calcification of chondrocytes in the growth plate in mice. Therefore, it provides us the opportunity to investigate the mechanism of postnatal bone growth.

Axis inhibition protein (Axin1) is the scaffold protein and belongs to the degradation complex which controls β-catenin degradation. As a negative regulator of β-catenin signaling, Axin1 interacts with other members of the degradation complex to phosphorylate and ubiquitinate β-catenin protein in the cytoplasm. Blocking the translocation of β-catenin from the cytoplasm to the nucleus could interrupt β-catenin interaction with transcription factors. These transcription factors, including T cell factor and lymphoid enhance factor, are responsible for the activation of β-catenin target genes. Activation of β-catenin signaling in cartilage tissue promotes chondrocyte maturation and perichondrial bone formation through a bone morphogenic protein 2 (BMP2)-dependent mechanism. BMP2 plays important roles in skeletal development and continuous postnatal bone growth.^3^ β-catenin could act as an upstream modulator of BMP2 in regulating osteoblast proliferation and differentiation. Fibroblast growth factor (FGF) has been suggested to regulate chondrocyte function. More specifically, abnormal FGFR3 expression was suggested to be involved in chondrodysplasia with disorganized chondrocyte columns in transgenic mice.

In this study, we deleted *Axin1* in aggrecan-expressing cells at postnatal and adult stages. We bred *Axin1*^*flox/flox*^ mice^4^ with *Agc1-CreER* mice to produce *Axin1*^*Agc1ER*^ KO (*Axin1* cKO) mice. We applied tamoxifen to two-month-old *Axin1* cKO mice and sacrificed mice at the age of six months. We first performed immunohistochemical analysis and demonstrated that Axin1 expression was significantly reduced in the growth plate and articular cartilage tissues (Fig. 1A, B). Consequently, β-catenin expression was up-regulated (Fig. S1). Histology analysis showed significantly disorganized and enlarged growth plate cartilage (Fig. 1C). The proliferating and hypertrophic chondrocytes lose their columnar structure and alignment parallel to the vertical axis (Fig. 1C). Micro-CT analysis showed bone mass increase phenotype in *Axin1* cKO mice (Fig. 1D). Results of quantification analysis demonstrated that the bone volume was higher in *Axin1* cKO mice compared with their Cre-negative littermate controls (Fig. 1E). Another index such as trabecular number (Tb.N.) (Fig. 1F) was increased while trabecular separation (Tb.Sp.) and structure model index (SMI) were decreased in *Axin1* cKO mice (Fig. 1G, H). The results of RNA (real-time PCR assay) and protein (immunohistochemical assay) expression analyses showed that expression levels and Col-X- and MMP13-positive cell numbers were significantly increased in *Axin1* cKO mice (Fig. 1I–N), further indicating that the process of chondrocyte hypertrophy was accelerated in *Axin1* cKO mice. We then analyzed changes in BMP signaling genes and found that expression of *Bmp2*, *Smad1*, and *Smad5* was significantly up-regulated in *Axin1* cKO mice (Fig. 1O–Q). We also analyzed the mRNA expression of *Fgfr2* and *Fgfr3* and found that both genes were up-regulated in *Axin1* cKO mice (Fig. 1R, S). In previous studies, we showed that *Fgfr3* expression was involved in the chondrocyte columnar disorganization in an osteoarthritis mouse model.^5^ We then examined changes in FGF downstream molecules, *Erk2* and pErk, and found that Erk2 and pErk expression were increased in *Axin1* cKO mice (Fig. 1T, U). Finally, we performed TRAP staining and quantified osteoclast numbers and *Opg* expression. We found that osteoclast formation was significantly decreased and *Opg* expression was significantly increased in *Axin1* cKO mice (Fig. 1V–X). This reduced osteoclast formation may contribute to the phenotypes of high bone mass and expanded growth plate cartilage due to defects of removal of calcified cartilage in the growth plate in *Axin1* cKO mice.

**Figure 1:**
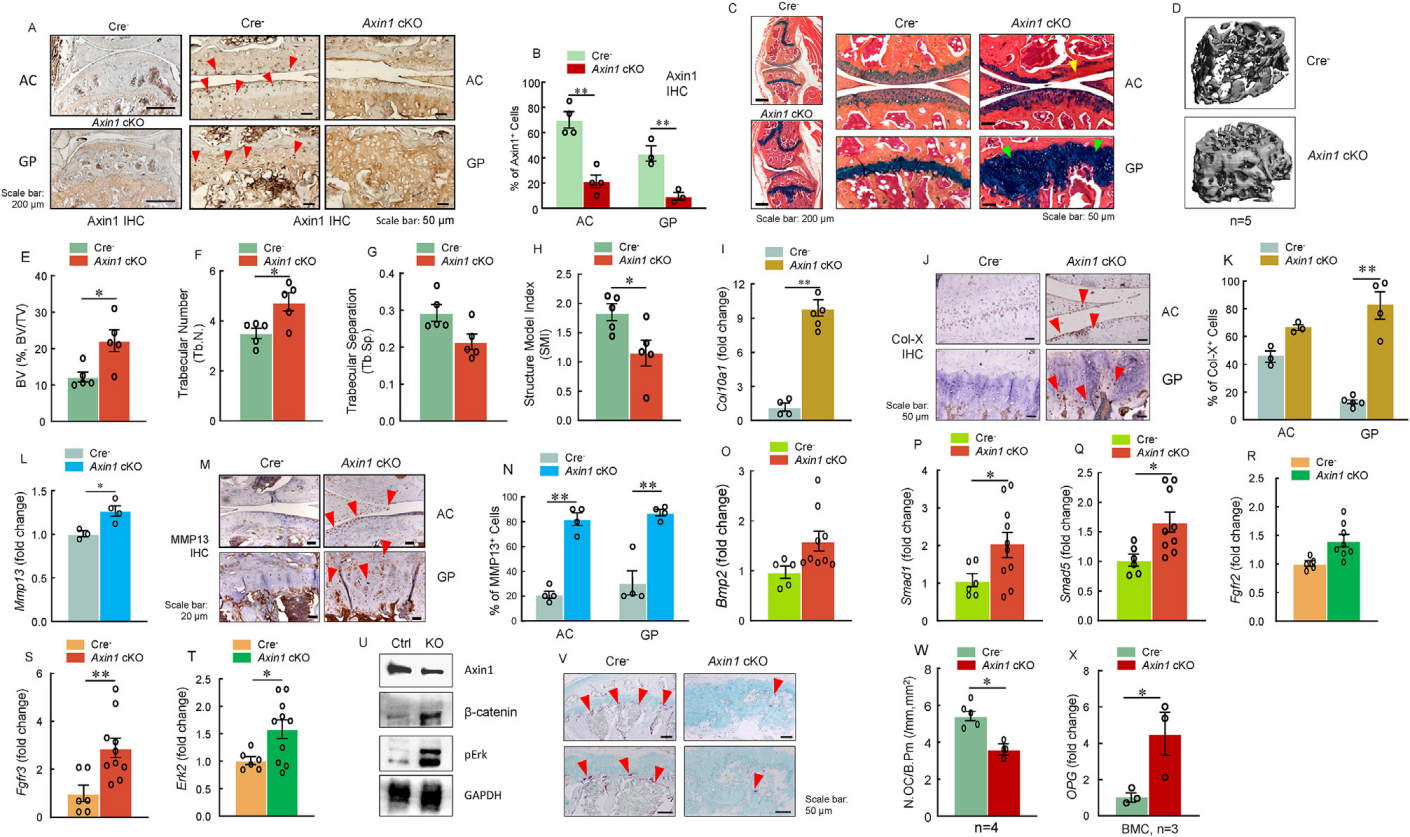
Deletion of *Axin1* in aggrecan-expressing cells leads to growth plate cartilage expansion and increased bone mass in *Axin1* cKO mice. **(A**–**C)** Immunohistochemical analysis showed that Axin1 expression was significantly reduced in joint tissues of six-month-old *Axin1* cKO mice. Red arrowheads, Axin1-positive cells; middle and right panels, higher magnification images; AC, articular cartilage; GP, growth plate. **(D)** Histology analysis (H&E staining) showed that the growth plate was disorganized and expanded (green arrowheads). In contrast, minor defects in articular cartilage degradation were also observed in six-month-old *Axin1* cKO mice (yellow arrowhead). **(E**–**I)** Micro-CT analysis showed bone mass was increased. Bone volume and trabecular numbers were significantly increased and trabecular separation and structure model index were significantly decreased in *Axin1* cKO mice. **(J**–**L)** Col-X mRNA expression and Col-X-positive cell numbers (red arrowheads) were significantly increased in six-month-old *Axin1* cKO mice. **(M**–**O)** MMP13 mRNA expression and MMP13-positive cell numbers (red arrowheads) were significantly increased in four-month-old *Axin1* cKO mice. **(P–U)** Real-time PCR analysis showed that expression of *Bmp2*, *Smad1*, *Smad5*, *Fgfr2*, *Fgfr3*, and *Erk2* mRNAs was significantly increased in chondrocytes derived from four-day-old *Axin1* cKO mice. **(V)** Western blot analysis was performed and β-catenin and pErk expression were up-regulated in *Axin1* cKO mice. **(W**–**X)** TRAP staining data showed osteoclast numbers were significantly reduced in six-month-old *Axin1* cKO mice (red arrowheads). In contrast, *Opg* mRNA expression was significantly increased in *Axin1* cKO mice.

During skeletal development, chondrocyte hypertrophy is a common process for endochondral ossification.^1^ Osteoblasts are then brought in during vascular invasion after removal of calcified cartilage by osteoclasts to form primary and secondary ossification centers.^1^ The resting, proliferating, and hypertrophic zones of the growth plate are contributing to bone growth and lengthening.^1^ Axin1 is a known scaffolding protein involved in the degradation process of β-catenin protein. We found that the growth plate region is dramatically expanded in adult *Axin1* cKO mice. In the growth plate, unlike Cre-negative littermates, both proliferating and hypertrophic chondrocytes were expanded to a great extent when *Axin1* was deleted associated with losing their alignment of directional columnar structure. These findings suggest that growth plate chondrocytes are more susceptible to the absence of Axin1. Col-X and MMP13 are hypertrophic chondrocyte markers. Increased Col-X and MMP13 expression were observed in the expanded growth plate of *Axin1* cKO mice. These findings are different from those observed in *Mmp9* and *Mmp14* KO mice. For example, the hypertrophic region was dramatically expanded in *Mmp9* KO mice (Suppl Ref 7), suggesting the critical role of MMP9 in hypertrophic zone remodeling during endochondral bone formation. Deletion of *Mmp9*/*Mmp14* in osteoclasts inhibited osteoclast formation (Suppl Ref 8) and defects in osteoclast formation also led to the expansion of the hypertrophic zone (Suppl Ref 9).

The synergistic effects between β-catenin and BMP are well documented in previous studies. It has been reported that fibroblast growth factor receptor (FGFR), especially FGFR3, was up-regulated in the cells in proliferating zone, while FGFR1 is mainly expressed by the cells in both pre-hypertrophic and hypertrophic zones. In our previous studies, we demonstrated that pErk expression was up-regulated in temporomandibular joints of *Axin1* cKO mice (Suppl Ref 10). Our findings suggest that in addition to the activation of BMP signaling, deletion of *Axin1* could also facilitate chondrocyte proliferation and hypertrophy through up-regulation of FGF signaling.

## Ethics declaration

The animal protocol used in this study has been approved by the IACUC of Shenzhen Institute of Advanced Technology (SIAT) and all procedures and methods were performed in accordance with the approved guidelines.

## Author contributions

The study was conceptualized by DC. Data curation and formal analysis were conducted by DY and HJ. DC and LT contributed to funding acquisition, project administration, and supervision, and wrote the original draft manuscript. WWL, GX, and CZ reviewed and edited the manuscript. All authors read and approved the manuscript.

## Conflict of interests

The authors of this paper declare that no competing interests are involved for all of them in this study.

## Funding

This project was funded by the 10.13039/501100012166National Key Research and Development Program of China (No. 2021YFB3800800) to LT and DC, the 10.13039/501100001809National Natural Science Foundation of China (NSFC; No. 82030067, 82161160342, 82250710174) to DC, and the Hong Kong RGC grant (HKU-17101821) to WWL and DC.
