# Supplementary material for: Deletion of Axin1 in aggrecan-expressing cells leads to growth plate cartilage defects in adult mice
Source: Genes Dis. 2023 Oct 19;11(4):101147. doi: 10.1016/j.gendis.2023.101147 (PMC10955195; doi:10.1016/j.gendis.2023.101147)
Supplement: Multimedia component 1 [file mmc1.doc]

### Supplementary data

**Deletion of *Axin1* in aggrecan-expressing cells leads to growth plate cartilage defects in adult mice**

**MATERIALS and METHODS**

**Generation of *Axin1* cKO Mice**

Axin1^f/f^ mice have been generated and used in our previous studies.^1^ *Agc1-CreER* mice^2^ were kindly provided by Jackson Laboratories (Bar Harbor, ME, USA). We bred *Axin1^flox/flox^* mice with *Agc1-CreER* mice to produce *Axin1^Agc1ER^* conditional KO mice (*Axin1* cKO). The Cre^-^ mice of the littermates of *Axin1* cKO mice were used as controls. Tamoxifen (Sigma, St. Louis, MO, USA) (0.7 mg per 10 g body weight) was given to the 2-month-old mice by intraperitoneal (i.p.) injection for 5 days. Mice were then euthanasia at 6-months of age. The animal protocol used in this study has been approved by Shenzhen Institute of Advanced Technology (SIAT) and all procedures and methods were performed in accordance with the approved guidelines.

**Micro-Computed Tomography (μCT)**

We fixed mouse legs with 10 % neutral buffered formalin (VWR, Radnor, PA, USA). μCT analysis was performed using the μCT-35 cone-beam scanner (Scanco Medical) with a 55 kVp source and a 145 μAmp current. The mouse legs were scanned at a resolution of 10 μm. Morphometric analysis was performed on 100 slices extending proximally, beginning with the first slice in which the tibia condyles had fully merged. The same thresholds were used to evaluate the scanned images to allow 3-dimensional structural rendering of each sample.^3^

**Histology and Immunohistochemistry**

We dissected knee joints from *Axin1* cKO mice and Cre-negative control mice. Samples were fixed in 10 % neutral buffered formalin (VWR, Radnor, PA, USA) for 3 days, then decalcified with formic acid (Decal Chemical Corp., Suffern, NY, USA) for 21 days and then paraffin embedded and were cut from the medial compartment of the knee joint. Histology staining was performed using Alcian Blue/Orange G staining method. IHC was performed on the 3 μm thick tissue sections^4^. 1:1000 dilution of Col-X (23419, Calbiochem, Germany), and 1:200 dilution of MMP13 (ab39012, Abcam, Cambridge, UK) were used in IHC assays.^3-5^

**TRAP Staining**

TRAP staining was performed on the 3 μm thick tissue section. Slides were incubated with TRAP staining buffer (Na acetate, Na tartrate, Fast Red Violet LB salt, Naphthol AS-MX, Dimethylformamide, MnCl_2_) for 1h at 37^o^C in the dark and then counterstained with CAT hematoxylin (Biocare Medical, CATHE-GL) and then coversliped with glycerol gelatin (GG1-15ML, Sigma, MO, USA). Quantification of osteoclast numbers (N.Oc/B.Pm) was performed with OsteoMeasure software (OsteoMetrics, Inc., Atlanta, GA, USA).

**Cell Culture and Real-Time Polymerase Chain Reaction (RT-PCR)**

Primary chondrocytes were isolated from articular and rib cage cartilage tissues of 4-day-old neonatal mice, as previously described.^6^ The isolated cells were cultured at 37^o^C with 5% CO_2_ in Dulbecco's modified Eagle's medium (DMEM) with 10% fetal bovine serum. 4-Hydrooxyfamoxifen (4-OH Tamoxifen, 1 μM, Sigma, St Louis, MO, USA) was added to the cell culture (24h culture) to induce *in vitro* *Axin1* gene deletion. Total RNA was extracted with Trizol (Invitrogen Life Technologies, CA, USA) and total RNA (1 μg) was used in the complementary DNA (cDNA) synthesis. iScripts cDNA Synthesis kit (Quanta Biosciences, MD, USA) was used in this assay. RNA amplification was conducted by real-time PCR using specific primers (Table 1) and SYBR Green real-time PCR kit (Quanta Biosciences, MD, USA).^3^

**Statistical Analysis**

The data were expressed as mean ± standard error. We used unpaired Student *t*-test to compare two groups of data and use one-way analysis of variance (ANOVA) followed by Turkey’s post-hoc test to compare data with multiple groups. **P* < 0.05 and ***P* < 0.01 are considered as significant difference between groups.

**References**

1. Xie R, Jiang R, Chen D. Generation of Axin1 conditional mutant mice. Genesis. 2011;49(2):98-102.
2. Henry SP, Jang CW, Deng JM, Zhang Z, Behringer RR, de Crombrugghe B. Generation of aggrecan-CreERT2 knockin mice for inducible Cre activity in adult cartilage. Genesis. 2009;47(12):805-14.
3. Liao L, Zhang S, Gu J, Takarada T, Yoneda Y, Huang J, et al. Deletion of Runx2 in articular chondrocytes decelerates the progression of DMM-induced osteoarthritis in Adult Mice. Sci Rep. 2017;7(1):2371.

4. Hui T, Zhou Y, Wang T, Li J, Zhang S, Liao L, et al. Activation of beta-catenin signaling in aggrecan-expressing cells in temporomandibular joint causes osteoarthritis-like defects. Int J Oral Sci. 2018;10(2):13.

5. Wang M, Li S, Xie W, Shen J, Im HJ, Holz JD, et al. Activation of β-catenin signalling leads to temporomandibular joint defects. Eur Cells Mater. 2014;28:223-35.

6. Gosset M, Berenbaum F, Thirion S, Jacques C. Primary culture and phenotyping of murine chondrocytes. Nat Protocols. 2008;3(8):1253-60.

7. Vu TH, Shipley JM, Bergers G, Berger JE, Helms JA, Hanahan D, et al. MMP-9/gelatinase B is a key regulator of growth plate angiogenesis and apoptosis of hypertrophic chondrocytes. Cell. 1998; 93: 411-422.

8. Zhu L, Tang Y, Li X, Kerk SA, Lyssiotis CA, Sun X, et al. Proteolytic regulation of a galectin- 3/Lrp1 axis controls osteoclast-mediated bone resorption. J Cell Biol. 2023;222(4):1-25.

9. Xing L, Chen D, Boyce BF. Mice deficient in NF-κB p50 and p52 or RANK have defective growth plate formation and post-natal dwarfism. Bone Res. 2013;4:336-345.

10. Xing L, Chen D, Boyce BF (2013) Mice deficient in NF-κB p50 and p52 or RANK have defective growth plate formation and post-natal dwarfism. Bone Res**.** 2013;1(4):336-345.

Table1. The names of sequences of primers used in this study

| Genes | Primer sequence (forward primers) | Primer sequence (reverse primers) |
| --- | --- | --- |
| Col-X | TTCTGCTGCTAATGTTCTTGACC | GGGATGAAGTATTGTGTCTTGGG |
| Mmp13 | CTTCTTCTTGTTGAGCTGGACTC | CTGTGGAGGTCACTGTAGACT |
| Bmp2 | TGCACCAAGATGAACACAGC | GTGCCACGATCCAGTCATTC |
| Smad1 | CGTCCAACAATAAGAACCGCTT | CACGGATGAAATAGGATTGTGG |
| Smad5 | ATGCCCAGCATATCCAGCAG | CAGAAGAAATGGGGTTCAGC |
| Ffgr2 | TCGCATTGGAGGCTATAAGG | CGGGACCACACTTTCCATAA |
| Fgfr3 | GCATCCTCACTGTGACATCAAC | CCTGGCGAGTACTGCTCAAA |
| Erk2 | CGCTTCAGACATGAGAACATC | GGTCCGTCTCCATGAGGT |
| β-Actin | GGCTGTATTCCCCTCCATCG | CCAGTTGGTAACAATGCCATGT |


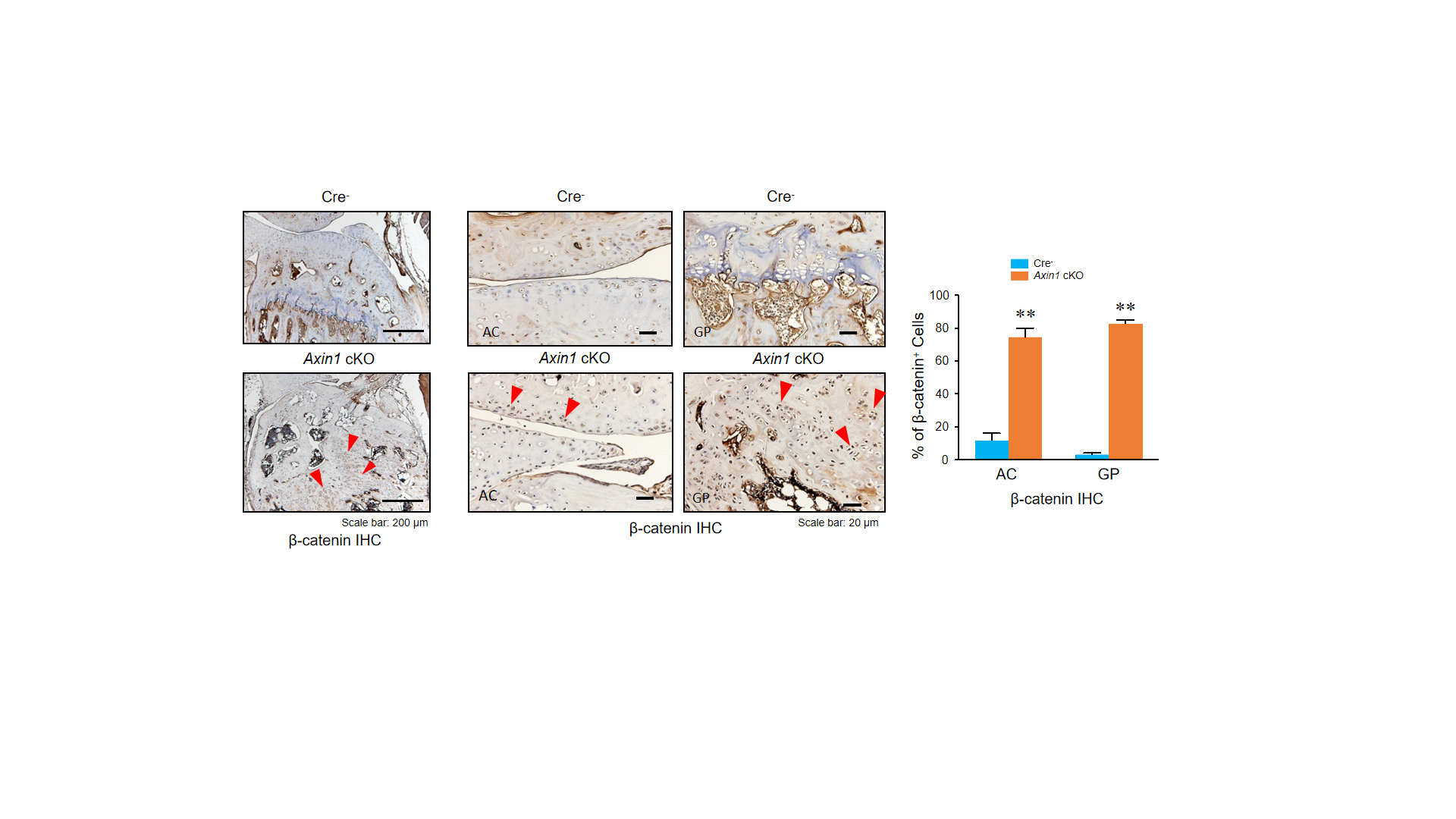


**Figure S1** Immunohistochemistry (IHC) analysis showed that β-catenin expression was significantly upregulated in joint tissues of 6-month-old *Axin1* cKO mice (red arrowheads: β-catenin-positive cells). Right side bar graph: quantification of β-catenin-positive cells in articular cartilage (AC) and growth plate cartilage (GP).
